# Biological and ecological traits of marine species

**DOI:** 10.7717/peerj.1201

**Published:** 2015-08-18

**Authors:** Mark John Costello, Simon Claus, Stefanie Dekeyzer, Leen Vandepitte, Éamonn Ó Tuama, Dan Lear, Harvey Tyler-Walters

**Affiliations:** 1 Institute of Marine Science, University of Auckland, New Zealand; 2 Vlaams Instituut voor de Zee, VLIZ–InnovOcean site, Oostende, Belgium; 3 Global Biodiversity Information Facility, Copenhagen, Denmark; 4 Marine Biological Association, Plymouth, Devon, UK

**Keywords:** Taxonomy, Distribution, Feeding, Diet, Body-size, Life-history, Habitat, Environment, Databases, Depth

## Abstract

This paper reviews the utility and availability of biological and ecological traits for marine species so as to prioritise the development of a world database on marine species traits. In addition, the ‘status’ of species for conservation, that is, whether they are introduced or invasive, of fishery or aquaculture interest, harmful, or used as an ecological indicator, were reviewed because these attributes are of particular interest to society. Whereas traits are an enduring characteristic of a species and/or population, a species status may vary geographically and over time. Criteria for selecting traits were that they could be applied to most taxa, were easily available, and their inclusion would result in new research and/or management applications. Numerical traits were favoured over categorical. Habitat was excluded as it can be derived from a selection of these traits. Ten traits were prioritized for inclusion in the most comprehensive open access database on marine species (World Register of Marine Species), namely taxonomic classification, environment, geography, depth, substratum, mobility, skeleton, diet, body size and reproduction. These traits and statuses are being added to the database and new use cases may further subdivide and expand upon them.

## Introduction

World databases of marine species have now been published but are limited to taxonomic (e.g., WoRMS) and distribution (e.g., Ocean Biogeographic Information System, OBIS) data (Costello et al., 2007). The benefits of these databases could be multiplied by associating species with richer ecological and biological information. Classification of species provides hypotheses for the evolution, organisation, and ecological interactions of biodiversity from genes to ecosystems. Initially, newly discovered species are classified by their taxonomic relationships, which are intended to indicate their evolutionary lineages and origins. New research challenges this classification, resulting in changes to species genera and even changes in higher taxonomic classification (Costello et al., 2013). Species are readily classified by their geography, for example what region, country or locality they occur in, and within that, by environment (e.g., freshwater, terrestrial, marine, soils or sediments). Ecological classification is more complex, and may refer to their habitat, a concept combining the physical environment and associated species with which the species typically occurs (Costello, 2009). Species may be associated with a guild of co-occurring species similar in distribution and habit; such as benthos, plankton, sessile epifauna, or ectoparasites. Biological classification includes attributes of life stages, reproduction, body size, behaviour, feeding method, and diet. However, data on such attributes or species traits are widely scattered in the literature and are time consuming to gather (Naeem & Bunker, 2009; Tyler et al., 2012). To solve this, databases of traits for (a) 21,000 species of freshwater plants, invertebrates and fish in Europe (Schmidt-Kloiber & Hering, 2015), and (b) terrestrial plants (Naeem & Bunker, 2009; Kattge et al., 2011), have been established.

A rich terminology surrounds descriptions of a species biology and ecology, with sometimes different definitions for the same terms, synonymous terms, and context dependent (e.g., habitat) terms (e.g., Lepczyk, Lortie & Anderson, 2008). This terminology has developed over several hundred years of natural history, in different languages, and often terms have multiple meanings in common use. For example, “littoral” habitat can be the marine zone between the low and high tide marks, extend to the continental shelf and include coastal river catchments, and refer to the edge of freshwater lakes (Aquatic Sciences and Fisheries Abstracts, 2014). The lack of standard use of terms can compromise the bringing together of this knowledge from different sources, and thus limit understanding of patterns beyond local scale, context specific studies (Lepczyk, Lortie & Anderson, 2008). Hence the publication of a glossary particular to the marine biology community (Costello et al., 2010) that followed a popular biology and ecology dictionary (Lincoln, Boxshall & Clark, 1998). That glossary provided the starting point for a standard vocabulary to be used in the World Register of Marine Species (WoRMS) database (Costello et al., 2013).

In this paper, we review and classify traits so as to decide which should be prioritised to apply to marine species in WoRMS. In parallel, we are developing a wider vocabulary and classification of traits that would provide the basis for expanding the traits in WoRMS in the longer-term. Thus, scientists interested in more detailed trait classifications for a particular taxon or ecological function could build on the more general primary traits proposed here.

### Biodiversity databases

Global databases that integrate information on species force the development of standard classifications (Costello & Vanden Berghe, 2006). This process then enables analyses across many species and datasets previously compromised by inconsistent terminologies. The World Register of Marine Species (WoRMS) is such a database (Costello et al., 2013; Boxshall et al., 2014). It contains the names of almost all known marine species and classifies them (1) taxonomically, (2) by environment (e.g., marine, freshwater, terrestrial), and (3) by geographic distribution. Each additional field in the database may have a multiplier effect on how useful the database may be to researchers, educators and other users. For example, the availability of the author and year of description of each species, and their synonyms, has facilitated research into the rate of discovery of marine species (Costello & Wilson, 2011; Mora et al., 2011; Appeltans et al., 2012; Costello, Wilson & Houlding, 2012; Costello, Wilson & Houlding, 2013a; Costello, May & Stork, 2013b; Costello, May & Stork, 2013c; Costello, Houlding & Wilson, 2014a; Costello, Houlding & Joppa, 2014b; Costello, Vanhoorne & Appeltans, 2015).

Several databases already include information on marine species traits, namely WoRMS, BIOTIC (Marshall et al., 2006), FishBase (Froese & Pauly, 2014), and SeaLifeBase. The SeaLifeBase data fields are a subset of those in FishBase. These databases have already applied some traits to marine species and it can be preferable to build on these applications than start anew. However, they contain hundreds of traits which would take considerable effort and resources to apply to all marine species. Thus, in this paper we present a rationale for the prioritisation of traits for immediate inclusion in WoRMS.

### User needs

Particular groups of users have begun to develop thematic databases within WoRMS. For example, species involved in Harmful micro-Algal Blooms (HAB) (Moestrup et al., 2013), occurring in the deep sea (WoRDSS, Glover, Higgs & Horton, 2013), and that have been introduced by human activities (Pagad et al., 2015). Biological traits may also be used to help predict a species sensitivity to toxic substances (Baird & Van den Brink, 2007), but may be a poor predictor of its likelihood of going extinct, becoming invasive, and/or its reaction to climate change (Angert et al., 2011). However, a failure to detect which traits affect a species’ ecology at a global level may be because traits are operational within a local and regional context (Vermeij & Leighton, 2009). That is, the importance of traits is relative to the ecological and environmental factors acting on individuals of a species at any time.

Traits that determine ecological function can be better predictors of invasiveness of marine fouling communities (e.g., Atalah, Costello & Anderson, 2007; Atalah et al., 2007; Wahl et al., 2011) and be less sensitive to sampling effort for sediment macrobenthos (Törnroos & Bonsdorff, 2012) than taxonomic richness. The richness of traits in an assemblage of species is positively correlated with species richness but not necessarily linearly (e.g., Cumming & Child, 2009; Törnroos & Bonsdorff, 2012). Other users of marine species data include ecologists studying the functional role of species in ecosystems (e.g., Naeem & Bunker, 2009; Boström, Törnroos & Bonsdorff, 2010). They may wish to know a species’ place in the food web and body size. The value of biodiversity to society is being quantified in terms of ecosystem goods and services, with the species’ importance being dependent on their functional role in the ecosystems. Conservation biologists conduct species extinction risk assessments using standard criteria based on species biological (e.g., population size and trends, generation time, age at maturity, longevity, fecundity, natural mortality) and geographic (e.g., range size) traits IUCN, 2012; Grave et al., 2015). Invasive species are an increasing concern. So information on which species have been introduced beyond their native range by human activities and have become invasive is in demand (Blackburn et al., 2014; Jeschke et al., 2014). Whether a species is likely to be transported by human activities, such as in ballast water, fouling on a ship-hull or aquaculture equipment, may depend on its habitat, habit and modes of dispersal (Brine, Hunt & Costello, 2013). Gallien, Carboni & Münkemüller (2014) proposed phenotypic similarity, based on taxonomic and functional traits, can predict invasiveness in communities.

We propose that the traits that users need should be prioritised for inclusion in databases. Ideally, this should result in users publishing new analyses resulting from the inclusion of traits in the database, which in turn would drive improvements in the quality and quantity of trait information. For the purpose of this paper, we identify two main classes of users, scientists (usually ecologists) and wider society. Ecologists require traits that identify a species’ role in an ecosystem. These traits provide the basis for understanding and assessment of species socio-economic importance. Society is interested in species by virtue of their importance as food (e.g., fisheries, aquaculture), threat to human and animal health (e.g., toxic algae and other species, sharks), pests (e.g., invasive), and likelihood of extinction.

It must also be recognised that most traits are not available in the literature for most species. For British North Sea macrobenthos, body size was the most available trait (Webb, Tyler & Somerfield, 2009). Tyler et al. (2012) found there was no trait data available for about a quarter of the North Sea macrobenthic species, and most traits were only available for about another quarter. They found that adult mobility, feeding method, development mode, sociability, migration and life span were available for only 30–40% of the species with body size data. The most valuable traits for end users wishing to compare traits across taxa will be those available for most species.

### Data

Data related to species may be of numerical, continuous and categorical form (Törnroos & Bonsdorff, 2012). Most traits are categorical, that is they are a concept described in a word that may or may not apply to a species, such as whether a species is a parasite or not. Törnroos & Bonsdorff (2012) show the utility of categorical traits for marine benthos because a wider variety of concepts and traits can be applied to species than if limited to numerical measures. However, some traits can be described by numerical data, such as body size and depth distribution, and geographic distributions by continuous variables such as contours on maps. Numerical and continuous trait data are preferable because they can be converted into categorical (concept-based) data but not the reverse. Thus, an actual depth range would be preferred to ‘bathyal’ or ‘mesopelagic’ because the latter categories cannot be converted to a depth range.

Most traits will need to be applied to a particular life stage, probably the adult stage in the first instance. In some cases traits may vary between sexes and populations (e.g., body size). Population level traits would require each trait to be placed in the context of the sampled location, and it may be unclear as to how representative they may be of the species.

### Aims

In this paper, we review traits assigned to marine species in existing biodiversity databases, and evaluate which would be most useful to users to prioritise for inclusion in WoRMS. The criteria for prioritisation were that (1) the trait could (in theory) be applied across most taxa, (2) that information on the trait existed for most taxa, and (3) it was likely that the availability of the trait would result in new uses of the databases in the short-term. As there are arguments for more and fewer traits depending on user needs, we created a top-10 shortlist. If a trait could be applied at a higher taxon level (e.g., family, order, phylum) this would make it easier to apply across many species. Where possible, we favoured numerical and continuous traits over categorical. Thus, although traits peculiar to populations rather than a species, and secondary traits derived from others, were not prioritized for inclusion in WoRMS, these are included in a wider classification and vocabulary for discussion by users.

## Methods

The prioritization of traits for marine species involved a review of the use of traits in literature and related databases, and asking experts in a range of taxa (including crustaceans, molluscs, fish, echinoderms, algae, birds, nematodes, annelids), and benthic and pelagic ecology of coastal and deep sea environments (listed in the Acknowledgements) their opinion on how to rank traits by importance and what uses they may make of an enhanced marine species trait database. Initial capture of potential traits and trait values made use of spreadsheets but as the development of a traits vocabulary is, of necessity, a community process involving discussion and feedback leading to consensus, the suitability of the open source Semantic MediaWiki (SMW) (https://semantic-mediawiki.org/) was investigated for building a hierarchical list of traits. SMW, an extension to MediaWiki, the wiki engine underlying Wikipedia, allows the content within wiki pages to be semantically marked up for subsequent processing and querying. It is well suited for capturing hierarchical knowledge organisation systems such as thesauri or other taxonomies. SMW is receiving some attention within biodiversity informatics having been adopted by Biowikifarm (http://biowikifarm.net/) which hosts several installations, e.g., for the TDWG draft standard, Audubon Core (http://terms.tdwg.org/wiki/Audubon_Core), with a dedication to long term sustainability through a consortium providing service sponsorship. SMW provides a number of advantages. Each term (i.e., concept or trait) can have its own web page where labels, definitions and examples can be presented. User friendly web forms can be used in place of raw wiki mark-up by domain experts to add content including translations to multiple languages. An associated discussion page allows capture of comments relating to a term so they are all conveniently available for review and building consensus. Relationships between terms can be established and the terms can be grouped into categories and collections. SMW can be scripted to output collections of terms in standard formats such as Resource Description Framework (RDF) (http://www.w3.org/standards/techs/rdf) and Simple Knowledge Organization System (SKOS) (http://www.w3.org/TR/skos-reference/) thereby making them more easily usable by other applications. Following best practices, SMW supports the issuing of resolvable identifiers for terms and the importing of already existing terms from other vocabularies so they can be re-used rather than re-invented.

For the marine species traits vocabulary, a customised version of SMW was established within the VLIZ hosted Coastal Wiki (http://www.coastalwiki.org). This wiki is an encyclopaedia providing up-to-date high quality information for coastal and marine professionals, which is continuously improved, complemented and updated by expert users. The wiki was implemented to allow collaborative writing by authors who can add new terms or improve and update existing articles. The main difference between this wiki and the online Wikipedia are the procedures to maintain the quality, consistency and comprehensiveness of the information (Claus et al., 2008). Within the wiki, an additional namespace, ‘*traits*’ was created. The namespace name is a variable for searching in, and reporting on, sets of pages. It is also used to apply features that configure the sets of pages in one namespace differently from another namespace. So every trait name, value, concept and collection falls under the namespace ‘traits’ within the Coastal and Marine Wiki, and is available under the same base URL as the World Register of Marine Species at http://www.marinespecies.org/traits/wiki. It is intended that the wiki will provide further functionality based on user feedback. Developing a hierarchy of traits, expressed formally in SKOS, will provide the foundation for future, semantically richer ontologies where a marine species traits ontology can draw on other published vocabularies and ontologies, including the Environment Ontology (http://environmentontology.org) and the Phenotypic Quality Ontology (PATO) (wiki.obofoundry.org/wiki/index.php/PATO).

## Results

### Traits in databases

Most of the traits in BIOTIC (Table 1) and FishBase (Table 2) can be applied to most marine species. The trait categories and descriptors used in BIOTIC were developed by the MarLIN project, with minor amendments (Hiscock, Jackson & Lear, 1999; Tyler-Walters et al., 2001). They encompass distribution, biology, phenotypic and genetic attributes, and importance to humans. FishBase has evolved over 20 years and is the most comprehensive database on any global taxon. However, in both databases there can be overlap between groups of traits, and some traits developed for particular use cases or projects at a level of detail would be impractical to achieve for most marine species in the short-term. For example, BIOTIC has separate classifications for habit, sociability, environmental position, growth form, mobility, dependency and host, which contain overlapping and/or strongly inter-dependent traits, and include bioturbation and fragility traits that are applicable to limited groups of species. Thus, it is necessary to review and select a simpler classification of traits in the first instance. Other classes of traits in BIOTIC include ‘Reproduction’ (regeneration, frequency, development mechanism, reproductive type), and ‘Distribution and Habitat.’ The latter includes: Migration Pattern, Biological Zone (depth zone categories), Physiographic features, Salinity, Substratum (includes biogenic habitats, crevices and sediment mixtures), Water Flow Rate, and Wave Exposure. In BIOTIC, body size data was available for *ca* 96% of the 685 species covered, but only half of the traits were complete for 60% of the species (Table 3).

**Table 1:** Benthic invertebrate traits in BIOTIC. List of benthic invertebrate traits compiled in the biological traits information catalogue BIOTIC (Marshall et al., 2006). Where more than one category of traits applies, all relevant categories are recorded.

Subject area	*Traits* (categories)
Biology	*Growth form*—44 categories e.g., Algal gravel, Bivalved, Foliose, Turbinate, Encrusting
	*Growth rate* (expressed as μm, mm, cm per day/month/year)
	*Size (max.)*—6 categories from Very small (<1 cm) to Large (>50 cm)
	*Environmental position*—14 categories e.g., Epibenthic, Infaunal, Interstitial, Pelagic, Demersal
	*Habit*—10 categories e.g., Attached, Bed forming, Burrow dwelling, Erect Encrusting
	*Height (above substratum)*—(mm/cm/m)
	*Flexibility*—High (>45°)/Low (10–45°)/None (<10°)
	*Fragility*—Fragile, Intermediary, Robust
	*Mobility/movement*—Swimmer, Crawler, Burrower, Drifter, Attached (permanent, temporary)
	*Dispersal potential (adult)*—7 categories from None, Very limited (<1 m) to >10 km
	*Feeding method*—19 categories e.g., Autotroph, Detritivore, Grazer, Predator
	*Typical food type* (descriptive text)
	*Bioturbator*—4 categories e.g., Diffusive mixing, Conveyor belt transport
	*Sociability*—Free living, Gregarious, Colonial
	*Dependency*—Independent, Parasitic, Mutualist, Inquilinist, Commensal, Host
	*Toxicity*—(Yes/No)
	*Host (for another species)*—(Yes/No)
Habitat	*Distribution (UK & Global)*—(descriptive text)
	*Biogeographic range*—(descriptive text)
	*Migratory*—Resident, Passive, Active (Diel, Seasonal)
	*Depth range* (expressed as metres below chart datum)
	*Substratum preferences*—38 categories, e.g., Bedrock, Boulders, Mud, Gravel, Mixed, Other
	*Physiography*—9 categories e.g., Open coast, Strait/sound, Sea loch, Ria/Voe, Estuary
	*Biological zone*—Benthic (15 categories), Pelagic (8 categories)
	*Wave exposure*—8 categories form Extremely Exposed, to Ultra Sheltered
	*Tidal strength*—Very Strong, Strong, Moderately Strong, Weak, Very Weak (negligible)
	*Salinity (range)*—Full (30–40 psu), Variable (18-40 psu), Reduced (18–30 psu), Low (<18 psu)
Life-history	*Reproductive type*—17 categories e.g., Budding, Fission, Gonochoristic, Hermaphrodite
	*Regeneration potential*—yes/no
	*Reproductive frequency*—7 categories e.g., Semelparous, Annual episodic, Biannual protracted
	*Reproductive season*—(range of months or seasons)
	*Reproductive location*—As adult, Adult burrow, Brooding, Sediment surface, Water column
	*Life-span (max.)*—8 categories from <1 year, to 100+ years
	*Generation time* 8 categories from <1 year, to 100+ years
	*Age at maturity*—8 categories from <1 year, to 100+ years
	*Fecundity*—number of eggs
	*Egg or propagule size*—value (μm, mm, cm)
	*Fertilization type*—External, Internal, Self-fertile, None (asexual)
	*Developmental mechanism*—10 categories e.g., Planktotrophic, Oviparous, Viviparous
Larval	*Larva dispersal potential*—7 categories from None, Very limited (<1 m) >10 km
	*Larval settlement period*—(range of months or seasons)
	*Duration of larval stage*—<1 day, 1 day, 2–10 days, 11–30 days, 1–2 months, 1–6 months, >6 months

**Table 2:** Traits in FishBase. A summary of traits included in FishBase (Froese & Pauly, 2014).

Taxonomy	Biology		Status
Common names	Age	Mass conversion	Introductions
Synonyms	Size	Metabolism	Aquaculture
	Growth	Diseases	Aquaculture profile
**Distribution**	Length–weight relationship	Fish sounds	Processing
Countries	Length–length	Gill area	
FAO areas	Length–frequencies	Otoliths	Genetics
Ecosystems	Morphometrics	Brains	Strains
Occurrences	Morphology	Vision	Allele frequencies
	Maturity	Swimming speed	Heritability
**Ecology**	Spawning	Swimming type	
Diet	Fecundity	Ecotoxicology	Stocks
Food items	Eggs	Ciguatera	Recruitment
Food consumption	Egg development		Abundance
Ration	Larvae		
Predators	Larval dynamics		
	Reproduction		

**Table 3:** Completeness of traits in BIOTIC. The completeness of trait information for species in BIOTIC (Marshall et al., 2006).

Trait	No. species	Percentage of species (*n* = 685)
Body-size	664	96.93
Mobility	407	59.42
Sociability	395	57.66
Feeding method	392	57.23
Habit	369	53.87
Fragility	366	53.43
Flexibility	363	52.99
Developmental mechanism	340	49.64
Regeneration	330	48.18
Reproductive type	322	47.01
Dependency	315	45.99
Growth form	302	44.09
Substratum	296	43.21
Food type	288	42.04
Distribution in UK	283	41.31
Depth range	283	41.31
Global distribution	282	41.17
Environmental position	282	41.17
Life-span	276	40.29
Reproductive season	272	39.71
Fertilization type	258	37.66
Reproductive frequency	254	37.08
Reproductive location	247	36.06
Maturity	236	34.45
Migratory	232	33.87
Larval settling time	230	33.58
Biological zone	221	32.26
Dispersal potential (Adult)	215	31.39
Salinity	212	30.95
Physiography	206	30.07
Dispersal potential (Larvae)	166	24.23
Wave exposure	166	24.23
Bioturbator	158	23.07
Egg size	158	23.07
Fecundity	155	22.63
Larval settlement period	148	21.61
Tidal strength	138	20.15
Generation time	136	19.85
Growth rate	115	16.79
Height	96	14.01
Biogeography	93	13.58
Toxic	50	7.30
Host	6	0.88

Taxon-specialist databases tend to contain traits that are difficult to apply to other taxa. For example, the TRY plant-trait database focuses on 52 groups of 681 traits characterizing the vegetative and regeneration stages of the plant life cycle, including growth, reproduction, dispersal, establishment and persistence (Kattge et al., 2011). These groups of traits were collectively agreed to be the most relevant for plant life history strategies, vegetation modelling and global change responses on the basis of existing shortlists and consultation with vegetation modellers and plant ecologists. Traits were summarized in groups, e.g., the group ‘leaf nitrogen content’ consists of the three traits: leaf nitrogen content per dry mass, leaf nitrogen content per area and nitrogen content per leaf. In the case of respiration, the database contained 105 related traits: different organs, different reference values (e.g., dry mass, area, volume, nitrogen) and the temperature dependence of respiration (e.g., Q10). Specific information for each trait is available on the TRY website (http://www.try-db.org). Previously, Cornelissen et al. (2003) proposed 30 functional “soft traits” for flowering plants for tackling large-scale ecological questions. These were grouped into vegetative (e.g., growth form, height, life span, phenology), regenerative (e.g., dispersal, seed size), leaf (e.g., size, nitrogen content), stem (e.g., density) and root (e.g., length, diameter, depth) traits. A study on bryophyte moss communities used metrics of plant size (i.e., shoot density, mass, height, surface area to volume ratio) (Michel et al., 2012). Traits common to all these databases were measures of growth form or habit, body size, longevity, nutrition, and dispersal mechanism.

## Prioritising Traits

### Distribution: environment, geography, depth, habitat, ecosystem, seascape

The term distribution may be applied to the environment and habitat in which a species lives, and its spatial distribution by geography, depth, and time. Temporal distribution is a numerical measure applied to particular traits, such as life span, duration of a life stage or time periods when a species changes its spatial distribution (e.g., population movement or migration). Thus, we do not propose it as a separate trait here because it can be included as a metric of traits.

#### Environment

In WoRMS, most species have already been attributed to one of the following environments: marine, brackish, freshwater, terrestrial, and combinations thereof (Table 4). When species are recognised as a host or parasite of one or more species they are then classified according to the environment of their host. A host may be considered the ‘habitat’ of a commensal species, including parasites and mutually beneficial symbiotic relationships (e.g., anemone fish). Many species change their habitat during different life stages, such as from planktonic larvae to benthic adults or parasites. Thus, a core attribute of a life stage is whether it is living in the pelagic or benthic environment. Pelagic may be sub-divided into pleuston, neuston, plankton (drifting), nekton, phyto-, zooplankton, demersal (= hyperbenthos, benthopelagic).

**Table 4:** Proposed priority traits for WoRMS. The 10 proposed priority traits and how they would be applied to adult marine species.

Trait	Relevance	Categories	Numerical
**1. Taxonomic**	Related species have similar traits so taxonomic relationships predict traits of related species	Kingdom to genus	Not applicable
**2. Environment**	Most studies are confined to a particular environment so this trait allows users to quickly isolate species of interest for their purpose.	Marine, brackish, freshwater, terrestrial, pelagic, benthic	Not applicable
**3. Geography**	Distribution is the most sought after information on species after its taxonomy.	Locality name	Latitude-longitude coordinates (in OBIS)
**4. Depth**	The most widely available variable to distinguish species’ habitat.	Intertidal, subtidal (epipelagic) deep-sea (>500 m)	Deepest and shallowest depth recorded in (1) literature and (2) in OBIS, above and below Chart datum (±m).
**5. Body-size**	Related to position in food web, species abundance, metabolic rates, and dispersal.	–	Maximum body length in mm excluding appendages. Maximum total body weight of individual.
**6. Substratum**	A key physical factor determining species habitat.	Sediment, hard, biological	Not applicable
**7. Mobility**	Indicates the dispersal potential of the life-stage.	Mobile, immobile (sessile)	Potential metres in life-time
**8. Skeleton**	Calcareous important for ocean acidification and fossil record. Gelatinous important due to sampling difficulties, role as predators, and hazard to humans.	Calcareous (aragonite, calcite), chitinous, silicious, exoskeleton, endoskeleton, plant cell wall	Not applicable
**9. Diet**	Influence on abundance of other species, determines position in food web.	Carnivore, herbivore, parasite, detrivore, phototrophic, chemoautotrophic	Isotopic signature Trophic level
**10. Reproduction**	May relate to the ability of a population to recover from reduced abundance or invisibility.	Sexual, asexual	

The occurrence of species in the fossil record has also been implemented in WoRMS. The indication of the fossil status of a taxon—Recent or Fossil or both Recent & Fossil—was found to be a necessity, as the type species of genera can contain extant taxa, extant species can be attributed to fossil species in taxonomic history and documenting fossil taxa can help prevent the accidental creation of junior homonyms. As the indication Recent and/or Fossil is too coarse for research questions involving evolution, phylogeny, biodiversity or biogeography, WoRMS is now also including detailed stratigraphic data. As WoRMS follows international standards on the level of taxonomy, it was decided to also follow the international standards for stratigraphy, by making use of the latest version of the hierarchically structured International Stratigraphic Chart (Cohen et al., 2013). Each stratigraphic range added to WoRMS is tied to a source, allowing traceability of information. As the hierarchy of the International Stratigraphic Chart is included, information can be added on the level available in the literature, and extrapolations can be made through the WoRMS search interface: e.g., all taxa appearing in a certain Age will automatically be included when searching for the corresponding Era or Epoch.

#### Geography

WoRMS utilises a gazetteer that enables species to be attributed to any predefined geographic area, including seas, oceans, and countries available at www.marineregions.org (Claus et al., 2014). Additional regions have also been identified for fisheries management and conservation reporting but are not presently included in WoRMS. Cross-mapping of geographic areas is possible to some extent. OBIS and the Global Biodiversity Information Facility (GBIF) provide actual latitude and longitude coordinates for over half of all marine species, often with place names and an indicator of geographic accuracy (e.g., 1 km^2^) (Costello & Wieczorek, 2014). They enable mapping of these locations as points, and from these geographic distribution can be inferred. Thus, through both the georeferenced place names in WoRMS and point locations in OBIS and GBIF, there are established methods to map marine species geographic distribution. Where distribution is not available as latitude–longitude coordinates, we recommend using the most geographically precise locality name possible; for example, ‘Dublin Bay’ should not be reported as the Irish Sea or north-east Atlantic.

#### Depth

There are several terms used to describe depth zones in the literature, although not with a consistently defined depth range (reviewed by Costello, 2009). Terms like neritic and oceanic, epipelagic, abyssal, and bathyal are concepts rather than strict depth zones. For example, the epipelagic is the zone with enough light for photosynthesis, and light penetration will vary with water clarity. Thus, photosynthesis occurs at greater depths in offshore waters than in more turbid coastal waters. If a species would have its deepest and shallowest known records reported, it could then be placed within any depth zone classification. The WoRMS deep sea database (Glover, Higgs & Horton, 2013) has chosen 500 m as the boundary for the ‘deep sea’ because below that temperature and light generally show little variation (Rex, 1981). A minimal depth zone classification could thus distinguish intertidal (or littoral, i.e., seabed exposed at low tide), subtidal (or sublittoral) and deep sea (>500 m depth) zones (Table 4). Beyond that it would be preferable to assign actual depth ranges from known data (e.g., from OBIS and literature).

#### Habitat

Habitat is highly context dependent and sometimes loosely applied. The term can sometimes be incorrectly used for a locality where a species occurs, or a seascape (e.g., bay, lough, estuary, island) which can contain a combination of habitats (Costello, 2009). However, in ecology a habitat is the physical environment in which a species lives at least part of its life. Many species change habitat during different life stages, such as from planktonic larvae to benthic adults or vice-versa. Habitats need to be distinguished from ecosystems and seascapes. The latter are defined by environment and geomorphology, and may contain any combination of benthic and pelagic habitats. They are now best mapped by remote sensing methods (e.g., acoustic, airborne, satellite) (Andréfouët et al., 2008; Costello, 2009).

A standard habitat and biotope classification was developed for European seas by the BioMar-LIFE project (Connor et al., 2004; Costello & Emblow, 2005) and subsequently expanded as part of the European Union Nature Information System (EUNIS) classification (Galparsoro et al., 2012). This is now well-established as part of the regulatory framework for nature conservation in Europe and its basic units of depth zonation, benthic substrata and wave and current exposure are common to other classifications. Its most detailed level describes a biotope, namely the physical habitat and associated community of species. A species may occur in more than one biotope. Some species define a biotope or habitat by virtue of providing a biogenic habitat within which other species live, such as reefs formed by corals, bivalves and worms, and beds of seaweed or seagrass. Thus, some species live in biological (biogenic) habitats, including symbionts and parasites. Matching each species to a biotope is possible where such ecological data are available. However, a simpler approach to characterise a habitat would be to record a species depth distribution, and if benthic, the substratum, or if biological, the host.

The simplest classification of benthic substrata would be sediment [i.e., mud (including silt), sand, gravel (including pebbles and cobbles), boulders] and hard substrata (e.g., bedrock, artificial substrata)(Table 4). As with environment, a species may occur in several of these (e.g., mud and sand, boulders and bedrock). A biological habitat could be subdivided into commensal, parasitic, and symbiotic. Thus the combination of depth, substratum and/or biological habitat (e.g., host if a parasite or symbiont, if associated with biological habitat), could be used to assign species to the habitat classification. A species’ abundance is likely to vary between habitats, and be facultative or obligate, such that it may occur in several which may make defining its habitat difficult. We recommend only assigning species to any habitat it is frequently found in. The small number of species limited to reduced or variable salinity (brackish and estuarine) habitats can be distinguished using the ‘environment’ classification. Thus we do not propose a separate trait called ‘habitat’ but rather users can derive it as appropriate to their needs using combinations of environment, depth and substratum (Table 4).

#### Ecosystem

The literature can often refer to species as being associated with habitats or geographic areas dominated or characterised by particular species, such as coral reef, seagrass or kelp ecosystems. Ecosystems are geographic areas defined by biologically significant environmental boundaries. Thus, they contain a diversity of habitats, and not the same proportion or combination of habitats in different areas. Because species are associated with habitats they are indirectly associated with ecosystems. Thus, it is difficult to assign an ‘ecosystem’ to a species. However, a species’ environmental limits can be defined and its geographic distribution can be mapped. Similarly, environmentally defined ecosystems may occur in different parts of the world but with different species. Thus, associating species with ecosystems is outside the scope of a species based classification. Rather, habitats could be mapped to ecosystems.

#### Seascape

Seascapes, sometimes called landscapes, geomorphological, topographic and physiographic features, are sometimes confused with habitats (Costello, 2009). However, while species are clearly associated with habitats, seascapes contain an idiosyncratic combination of habitats. Thus, like the situation with ecosystems, it is not necessary to assign species to seascapes because a coastal species, for example, may be associated with all potential seascapes depending on the habitats they contain. Thus, we consider seascapes outside the scope of a species classification. They may be applied when mapping habitats in particular geographic regions.

### Biological

#### Life stage

The traits of most marine species vary significantly between life stages. Most fish, crustaceans and molluscs have planktonic larvae but some cnidarians have pelagic adult stages. Thus, it is essential to qualify a trait by the life stage to which it applies. For some taxa, such as peracaridean crustaceans which brood their eggs and lack free living larvae, the traits may be the same for adults and juveniles. Thus, we propose four basic life stages: adult (mature), juvenile (immature but morphologically adult), larva (morphologically different from adult form), and egg (or propagule, spore). Some taxa have specific nomenclature for different life stages and multiple larval forms (e.g., nauplius, zoea, megalopa, phyllosoma, veliger) but these cannot be applied across all species. At present, we propose to prioritise traits for the adult life stage only because this is generally more available, can be applied to more species, and would be users’ first expectation.

#### Body size

Body size is perhaps the most fundamental trait as it correlates with other traits, for example, enabling conversion of length and abundance to biomass (e.g., Gifford & Caron, 2000; Postel, Fock & Hagen, 2000). In a review of 22 research areas using traits, body size was the most commonly used (Naeem & Bunker, 2009). It is also the most widely available trait (Table 3; Webb, Tyler & Somerfield, 2009; Tyler et al., 2012). Field sampling typically selects species based on body size, whether large enough to be identifiable on sight in the field, or if captured through nets (plankton) or sieves (benthos) of particular mesh size. Larger animal species tend to be top predators and smaller tend to be herbivores and/or detrivores, so body size correlates with food web structure, trophic levels, and energy flow in ecosystem (Gerlach, Hahn & Schrage, 1985). Some studies found peaks and troughs in body size distributions of benthic fauna (e.g., Gerlach, Hahn & Schrage, 1985). However, other studies did not, instead finding that size-distribution patterns reflected the species present rather than any habitat influenced structure (Dolbeth, Raffaelli & Pardal, 2014). Pelagic species have long been classified by body size because it is conveniently related to sampling method and can simplify data presentation and analysis (Platt & Denman, 1977). Nine classes of body length, each increasing by a factor of ten from 0.02 µm virio-plankton to 20 m nekton, are commonly used but this does not imply any ecological meaning to the size classes (Sieburth, Smetacek & Lenz, 1978). That said, viruses are all in the <0.2 µm size class; most bacteria in the 0.02–2.0 µm; most fungi, phytoplankton and protozoa spread across the next two (2–200 µm); and most metazoans are >0.2 mm (Sieburth, Smetacek & Lenz, 1978). There can be considerable size differences between larvae, juveniles and adults in metazoans; so a species may span several size classes.

Classifications based on body size such as macrobenthos, meiobenthos, and nekton, are for convenience rather than reflecting any true biological classification; so there is no a priori reason to place a whole species or life stage into a size class. We propose that this trait is defined as the typical maximum size reached by an individual of the species, be it body length, or diameter if circular (Table 4). The length of appendages, such as antennae, legs, wings, or tentacles, is excluded from ‘body length’ although, of course, may be included in taxon-specific traits. Some taxa may have additional length measurements to body length, such as wing span of birds, arms of octopuses, tentacles of jellyfish, and antennae of crustaceans. Thus, ‘body length’ of a coral’s body size will be that of its largest polyp (not the colony, if colonial), and an octopus’s length would exclude its arms. Where sexes differ in maximum body-size then the default would be the largest adult body length, but an additional field could be created where users wish to recognise differences between sexes. Similarly, traits could be associated with a geographic distribution where they vary sufficiently between populations. The maximum body weight for a species’ life stage can be more useful for studies on ecosystem energetics and should also be included where possible (Table 4). This would include its skeleton and thus its shell unless it was specified otherwise. The units may be wet weight or dry weight and need to be defined.

#### Life history

Traits describing the persistence of individuals and/or populations include growth rate and longevity (life span). Growth rate and age of maturation determine population generation time. The life span of individuals can indicate population stability over time and dispersal potential of various life stages (e.g., longer planktonic larva life span) and be measured in days, months and years. Fecundity indicates potential abundance, population productivity, and recovery from population decline, and can be measured as the number of eggs per female per spawning. Recruitment is the actual number of eggs surviving to become juveniles. However, most of these traits are only available for a few species and some are difficult to apply at a species level. Other biological traits can characterize the mode of reproduction of a species, such as whether ovoviviparous, viviparous, hermaphroditic, parthenogenic, asexual, protogynous, iteroparous or semelparous, involving brooding, nesting, or parental care. As a first step, we propose to distinguish species with sexual and asexual reproduction because such information is easily available for most taxa and may be significant with regard to the ability of a species to disperse, become invasive, and/or recover from a population decline. As with other traits, a species can be either or both.

#### Physiology

Species responses to climate change, particularly temperature rise and ocean acidification, will depend on their physiological tolerances. Thermal tolerance may be inferred from comparing species distributions to environmental data, such as conducted in species distribution modelling (e.g., Basher, Bowden & Costello, 2014). We do not prioritise the inclusion of experimental data because they will only be available for a small number of species. However, we see physiological traits as being of increasing interest and the availability of data should be reviewed in the future.

### Ecological

The three major classes of traits used in ecology relate to habitat, as covered previously, and habit and feeding. In ecology, habit is the external appearance or form of a species (Lincoln, Boxshall & Clark, 1998). Perhaps because more common usage refers to behaviour, this means a wide variety of traits have been related to habit. Habit is considered important because it can determine the mode of dispersal and ecological role (e.g., habitat forming) of species in an ecosystem. Rather than use the term, we propose to focus on the related trait categories of Mobility and Skeleton (Table 4). Species whose habit forms a physical habitat for other species are very important in ecology and often define ‘biogenic’ habitats. However, whether species form such habitats can depend on local conditions and abundance. Species may be colonial, tubicolous, encrusting, produce shells, or erect (e.g., seaweed) but they do not necessarily form reefs or forests. Future research needs to consider how to classify such variable attributes of species.

#### Mobility

The traits influencing a species dispersal potential tend to be encompassed by the growth form of individual animals (e.g., whether the life stage is mobile or sessile), abundance, and longevity. Dispersal of individual life stages is a variable of great interest regarding invasive species. However, it is rarely known from direct measurements and is estimated from observed colonization events. Thus, we do not propose a classification of dispersal per se but leave users to select traits that may influence dispersal of their taxa of interest. Instead, we propose a simple trait of mobility that can be scored as yes or no (if immobile) (Table 4), or ideally, assigned a distance of ‘ambit’ or dispersal potential (e.g., 0 m, <1 m if sedentary, >1 m, >10 m, etc.). All pelagic species will be classified as mobile by virtue of their medium, but only sessile benthic species as immobile (depending on their life stage). Where a species may be a host for a parasite or symbionts, then the latter is included in the trait ‘biological’ under substrata, and parasite under diet (Table 4).

Future development of this trait category may sub-divide it into sessile, sedentary, mobile (vagile, errant), solitary, aggregated, gregarious, fossorial, and interstitial. Aggregated could be sub-divided into schooling, swarming, and colonial (fixed together in colony). Mobile could be sub-divided into swimming, drifting (including rafting), crawling, burrowing, flying, gliding, and jet propulsion. Variants on these terms can be significantly different. For example, a species may live in burrows but not create them itself, so it is ‘burrow living’ but not fossorial.

#### Skeleton

The presence of hard skeletons, including shells, is an important factor in determining the fossil record of species. In addition, organisms with calcareous skeletons may be affected by ocean acidification. Ocean acidification is predicted to increase the physiological costs for species with calcareous skeletons and shells (Byrne, 2011; Byrne & Przeslawski, 2013), as it can impact marine organisms through a decreased calcium carbonate (CaCO_3_) saturation, thus affecting the calcification rates. The effect of this even increases at high latitudes and regions that intersect with pronounced hypoxic zones (Fabry et al., 2008), thus stressing the need to not only know whether a species has calcareous structures, but also to have information on its geographic distribution.

Many planktonic and benthic groups, such as Coccolithophora, Foraminifera, Pteropoda, Mollusca, Echinodermata, Crustacea, Cnidaria, Porifera, Bryozoa, Annelida, Brachiopoda and Tunicata—have CaCO_3_ skeletal elements. However, it is secreted under different forms: aragonite, calcite, high magnesium calcite, amorphous CaCO_3_ or a mixture of these phases (Mucci, 1983; Lowenstam & Weiner, 1989). Aragonite is about 50% more soluble in seawater than calcite (Mucci, 1983). Documenting the presence of a hard skeleton in combination with the present CaCO_3_ phase has been identified as a priority trait, as this can both be used in determining the fossil record of a species and its susceptibility to ocean acidification.

Many taxa lack calcareous skeletons. Diatoms have silica based skeletons, so availability of silica can affect primary productivity. Arthropods and some fungi have chitinous skeletons, while plants’ cell walls have a range of materials including cellulose and lignin. It may be important to users whether skeletons occur internally (e.g., fish) and/or externally to the body wall. Thus, we have prioritised four skeletal materials, calcareous, chitinous, silicious, and plant cell walls, and whether these form endo- or exo-skeletons (Table 4). Species without a hard skeleton can be so noted, as well. A considerable number of species lack such a skeleton, including worm-like taxa, gelatinous zooplankton, sea anemones, some molluscs (e.g., octopus, slugs). Gelatinous zooplankton, including jellyfish, salps and ctenophores, tend to be damaged and under-sampled by plankton nets. However, they are important predators, and some are hazardous to humans and can be considered pests. Based on the priority traits, a search of WoRMS on ‘pelagic’ and ‘skeleton absent’ will find soft-bodied plankton of which many could be considered gelatinous.

#### Diet and trophic level

Feeding can relate to either what a species feeds on, i.e., its diet if an animal, and/or how it feeds. Associated traits can become complex and species specific. We thus propose a simple classification of diet. We exclude scavenger because this is a behaviour rather than food type. Unless a food source is known it should not be assumed. Often, it is assumed that small invertebrates are omnivores or detritivores, when the actual importance of animal, plant and detritus in their diet is unknown, even if feeding has been observed. Some classifications include decomposers, but decomposition can be by a combination of carnivores or herbivores and microbial decay. Thus, it is covered by the other feeding categories and chemoautotrophs (heterotrophs).

We considered traits that described a species feeding method, such as particulate, suspension, deposit, filter and grazing feeding. These can be important in terms of classifying the functional role of species in an ecosystem. However, of greater importance is the trophic level a species occupies; that is, whether it is a detritivore, herbivore, primary, secondary or tertiary level carnivore. This can be inferred from the species diet and where available supported by isotope data (e.g., Heymans et al., 2014).

### Species’ importance to society

What users often wish to know is what the “status” of a species is with regard to its importance to society. This is not a fundamental trait of the species but reflects its current ‘status’ in some regard. This status may change over time, such as when a new fishery is established, a species becomes invasive, or it becomes more or less threatened with extinction. Thus, although the ‘status’ of a species is not a ‘trait’ as such, it is included in WoRMS. A species conservation status can be indicated by its inclusion in the IUCN Red List (IUCN, 2014), EU Habitats and Bird Directives (European Union, 1992; European Union, 2009), OSPAR List of Threatened and Declining Species and Habitats (OSPAR, 2008) and CITES (CITES, 2014) (Tables 5–7). The status of species known to cause Harmful Alga Blooms (HAB) is recorded within the WoRMS HAB Thematic Database (Moestrup et al., 2013). Species of importance for fisheries and aquaculture can be recognised by their listing in official catch statistics (Garibaldi & Busilacchi, 2002).

The IUCN Red List assessments require data on population trends in terms of abundance, natural mortality rates, and number of breeding individuals. Population–level are outside the scope of the present paper which concerns species level traits only. However, future classification could include traits related to fecundity, generation time, age at maturity, and geographic range, because these are used in the Red List assessments, and correlated traits such as maximum body size and age. These traits, plus growth rate and aggregation behaviour, also determine fish species susceptibility to overfishing (Morato, Cheung & Pitcher, 2006).

A further category that denotes societal importance of a species is its value as an indicator of ecosystem condition. The Marine Strategy Framework Directive is the key European marine environmental policy instrument. Its aim is ‘Good Environmental Status’ in European waters (MSFD 2008/56/EC). Good Environmental Status is divided into 11 descriptors, of which five are based on species composition: D1 biological diversity, D2 non-indigenous species, D3 commercial fish and shellfish, D4 food-webs, and D6 seafloor integrity. Once formalized, these status indicators, and equivalents for other regions of the world, will be added to the species in WoRMS.

Information on introduced species locations, dates recorded and population trends and impacts are required for management (Blackburn et al., 2014; Jeschke et al., 2014). This classification of species is the most difficult of all species attributes because of changing species status arising from misidentifications, and species becoming invasive in one place, perhaps temporarily, and not in others. Thus, there is a more complex terminology and structure required in the database which will be required to be described elsewhere. To date, the status of almost 1,400 introduced species has been recorded in WoRMS (Pagad et al., 2015).

At present, the conservation of marine species has been focused on chordates, including mammals, birds, reptiles and fish because these are most threatened with extinction (Tables 6 and 7). Of European marine species, the EU Bird and Habitats Directives list 100% of reptiles, 67% of lampreys, 65% of mammals, 61% of birds, 2% of fish, and <0.4% of all other taxa to be in need of protection (Table 5). Globally, the taxa with most endangered species are birds (26%), mammals (23%), reptiles (12%), and fish (3%). However, over 2% of cnidarians (hard corals) are considered endangered by IUCN and trade in 20% is restricted under CITES. Although 74% of marine mammal species are listed under CITES, only 9% of reptiles, 3% of birds and <1% of fish and other taxa (Table 6). The same higher taxa dominate species of economic importance as listed by FAO, namely (as a percentage of WoRMS): 76% mammals, 33% fish, 21% birds, 18% lampreys, and 14% reptiles. In contrast, introduced species are of very different taxa, namely 5% sipunculans, 3% entoprocts and tunicates, and 2% ctenophores, plants, and annelids (Table 5).

**Table 5:** Numbers of species in ERMS and WoRMS, and that are alien, cause HAB, and of conservation and economic importance. The number of species in higher taxa that occur in the European and World Registers of Marine Species (ERMS, WoRMS); are considered alien (=introduced) (or their origin in uncertain or unknown); been listed as of conservation importance by the European Union Birds or Habitats Directives; listed of regional ecological importance under the Oslo-Paris Convention (OSPAR); are associated with Harmful Algal Blooms (HAB); or are listed as being of international commercial fishery or aquaculture importance by the Food and Agricultural Organisation (FAO).

Taxon kingdom, phylum, or class	ERMS	WoRMS	Alien	Origin unknown	Origin uncertain	EU directive	OSPAR	HAB	FAO
Agnatha	6	93	0	0	0	3	0	0	17
Annelida	2,170	12,658	158	21	19	0	0	0	19
Aves	234	645	2	0	0	91	9	0	133
Bacteria	181	1,716	4	0	0	0	0	1	1
Bryozoa	800	6,112	58	4	3	0	0	0	0
Chaetognatha	41	131	1	0	0	0	0	0	0
Chelicerata	517	2,939	4	0	1	0	0	0	12
Chromista	3,929	20,285	172	26	1	0	0	115	42
Cnidaria	1,294	10,760	76	6	6	1	0	0	86
Crustacea	7,062	53,321	287	15	6	1	1	0	643
Ctenophora	39	187	4	0	0	0	0	0	1
Echinodermata	652	7,277	15	1	1	1	0	0	151
Echiura	37	197	1	0	1	0	0	0	0
Entoprocta	60	174	4	1	0	0	0	0	0
Fungi	399	1,363	8	0	0	0	0	0	0
Hexapoda	88	1,461	2	0	0	0	0	0	0
Mammalia	54	140	1	0	1	35	4	0	107
Mollusca	4,294	45,128	291	9	8	4	4	0	1,323
Nematoda	2,103	7,012	1	0	0	0	0	0	0
Pisces	1,451	17,858	206	3	6	28	22	0	5,892
Plantae	1,666	8,800	157	16	3	3	0	0	154
Platyhelminthes	2,133	12,134	16	2	3	0	0	0	0
Porifera	1,542	8,383	11	1	4	0	0	0	20
Reptilia	5	107	1	0	0	5	2	0	15
Rotifera	109	186	2	0	0	0	0	0	2
Sipuncula	42	147	7	0	0	0	0	0	2
Tunicata	495	3,031	59	20	1	0	0	0	24
**TOTAL**	33,149	227,585	1,548	125	64	172	42	116	8,644

**Table 6:** Number of species assessed for conservation concern. The number of species in higher taxa that had their conservation risk assessed on the global IUCN Red List as Extinct, Extinct in the wild, Critically Endangered, Vulnerable, Near threatened; or Least concern; and international trade restricted (listed by CITES). Taxa not represented in these categories were: Acanthocephala, Agnatha, Amphibia, Annelida, Brachiopoda, Bryozoa, Cephalochordata, Cephalorhyncha, Chaetognatha, Chelicerata, Ctenophora, Cycliophora, Dicyemida, Echiura, Entoprocta, Fungi, Gastrotricha, Gnathostomulida, Hemichordata, Hexapoda, Myriapoda, Myxozoa, Nematoda, Nemertea, Orthonectida, Phoronida, Placozoa, Platyhelminthes, Protozoa, Rotifera, Sipuncula, Tardigrada, Tunicata, Viruses, Xenacoelomorpha.

Taxon kingdom or phylum	Extinct	Extinct in wild	Critically endangered	Endangered	Vulnerable	Near threatened	Least concern	CITES
Chromista	0	0	4	1	1	0	0	0
Plantae	1	0	8	6	16	12	108	6
Porifera	0	0	0	0	0	0	0	0
Cnidaria	0	0	7	25	204	176	297	2,097
Mollusca	4	0	7	16	36	30	769	2
Crustacea	0	0	6	1	1	2	162	0
Echinodermata	0	0	0	0	9	1	111	1
Pisces	1	0	60	93	314	236	3,469	95
Reptilia	0	0	4	3	6	4	48	9
Aves	9	0	26	58	86	78	600	22
Mammalia	4	0	3	12	17	9	44	104
**TOTAL**	19	0	126	215	691	548	5,608	2,336

**Table 7:** Number of species in taxa not included in Tables 5 and 6. Number of species in taxa in the European and World Registers of Marine Species (ERMS, WoRMS) but not represented in any of the categories in Tables 5 and 6.

Taxon kingdom or phylum	ERMS	WoRMS
Acanthocephala	62	446
Amphibia	0	1
Archaea	–	119
Brachiopoda	39	395
Cephalochordata	2	30
Cephalorhyncha	62	236
Cycliophora	1	2
Dicyemida	17	122
Gastrotricha	256	491
Gnathostomulida	25	98
Hemichordata	17	130
Myriapoda	13	68
Myxozoa	212	473
Nemertea	378	1,359
Orthonectida	19	25
Phoronida	9	17
Placozoa	1	1
Protozoa	350	623
Tardigrada	83	170
Viruses	–	111
Xenacoelomorpha	200	423

## Discussion

Based on the criteria of applicability across most taxa, availability for most species, and potential usage, we prioritized 10 traits for inclusion in WoRMS (Table 4). Poelen, Simons & Mungall (2014) similarly prioritised taxonomy, environment, geographic location, altitude and depth, and functional group (e.g., planktonic) as proposed here. Taxonomy is already fully implemented, and the others partially. Indeed, as all traits are not available for all species their completion will be a continuing process. In addition, the conservation and introduced (potential pest) status of species will need to be regularly reviewed.

We see immediate applications for the traits. Research into species biogeography will be able to compare the distribution of taxa across ‘environments’ and depth gradients, and classify them by body size and trophic levels. OBIS uses WoRMS as their taxonomic standard and could also use the traits. Then OBIS users could select species not just by taxonomy but by their traits and, for example, conservation status or fishery importance. Ocean acidification studies will be able to compare the distribution of taxa with different skeletal composition. Paleontologists will be able to compare the species richness of taxa likely to be better preserved as fossils with taxa without durable skeletons. Gelatinous zooplankton occur in different phyla but could now be grouped by this trait. Analyses could test whether threatened, introduced and/or invasive species are a random subset of all marine species, or have particular traits that may predispose them to being threatened, introduced or becoming invasive respectively. For example, are mobile and/or asexual species more likely to be introduced, and less likely to be of conservation concern, because only one individual is required for dispersal?

Some users may be most interested in secondary traits, that is, traits dependent on combinations of the primary traits reviewed here. For example, bioturbation potential is predicted from a combination of known information for related species with regard to mobility, burrowing behaviour, biomass and abundance (Queirós et al., 2013). Dispersal potential may be predicted by combinations of mobility and environment (Angert et al., 2011). We understand some users will want additional sub-divisions of traits, for example, of salinity by estuarine ecologists (Reusser & Lee, 2011). The latter authors also sub-divided benthic, pelagic, and reproductive traits, but then combined environment, habitat, and seascapes, within a very broad definition of biogeography. Users that wish to implement specialist traits for a particular taxon are welcome to do so, and WoRMS is available to provide the infrastructure. If these are unique to the taxon then the development of such trait classifications is simplified. However, where they may overlap will require consideration by specialists on other taxa.

It is relatively easy to add more trait fields to a database. However, this can increase complexity, redundancy, duplication, and overlap between traits. We thus recommend that expansion of the trait classification in databases proceed cautiously and concisely, only adding traits with a proposed use and that are available for the taxa of interest.

## Additional Information and Declarations

### Data Availability

The following information was supplied regarding the deposition of related data: All data is publicly available in the World Register of Marine Species: http://www.marinespecies.org.
